# Traceable Calibration, Performance Metrics, and Uncertainty Estimates of Minirhizotron Digital Imagery for Fine-Root Measurements

**DOI:** 10.1371/journal.pone.0112362

**Published:** 2014-11-12

**Authors:** Joshua A. Roberti, Michael D. SanClements, Henry W. Loescher, Edward Ayres

**Affiliations:** 1 National Ecological Observatory Network (NEON), Boulder, Colorado, United States of America; 2 Institute of Arctic and Alpine Research (INSTAAR), University of Colorado, Boulder, Colorado, United States of America; DOE Pacific Northwest National Laboratory, United States of America

## Abstract

Even though fine-root turnover is a highly studied topic, it is often poorly understood as a result of uncertainties inherent in its sampling, *e.g.*, quantifying spatial and temporal variability. While many methods exist to quantify fine-root turnover, use of minirhizotrons has increased over the last two decades, making sensor errors another source of uncertainty. Currently, no standardized methodology exists to test and compare minirhizotron camera capability, imagery, and performance. This paper presents a reproducible, laboratory-based method by which minirhizotron cameras can be tested and validated in a traceable manner. The performance of camera characteristics was identified and test criteria were developed: we quantified the precision of camera location for successive images, estimated the trueness and precision of each camera's ability to quantify root diameter and root color, and also assessed the influence of heat dissipation introduced by the minirhizotron cameras and electrical components. We report detailed and defensible metrology analyses that examine the performance of two commercially available minirhizotron cameras. These cameras performed differently with regard to the various test criteria and uncertainty analyses. We recommend a defensible metrology approach to quantify the performance of minirhizotron camera characteristics and determine sensor-related measurement uncertainties prior to field use. This approach is also extensible to other digital imagery technologies. In turn, these approaches facilitate a greater understanding of measurement uncertainties (signal-to-noise ratio) inherent in the camera performance and allow such uncertainties to be quantified and mitigated so that estimates of fine-root turnover can be more confidently quantified.

## Introduction

The growth, senescence, and mortality of fine-roots, collectively referred to as fine-root turnover is key to estimate belowground nutrient and carbon cycling and ecosystem productivity [1], [2]. Production of fine-roots accounts for a third of the Earth's annual net primary production [3], supporting the notion that fine-root mortality returns more carbon and nitrogen to the soil than litterfall [1], [4]. Unlike aboveground primary production, fine-root production has only been measured at a limited number of sites and over relatively short timescales, in part, because of short-comings associated with sampling methods [5]–[7]. Currently, there is no standardized method or ‘best community practice’ for evaluating fine-root turnover [8], even though a number of approaches have been used in the past. For example, radiocarbon ^14^C measurements of fine-roots, although fundamentally sound in theory, provide estimates of mean carbon age based on modeled results that are often under question [9]. Methods such as sequential soil coring and ingrowth cores are reliable for quantifying the stocks of root biomass but embody large spatial and temporal uncertainties when assessing fine-root turnover as a result of sampling at different locations at each sampling event [8], [10], [11]. Moreover, root production and mortality of the same root size, and in some cases, species, cannot be observed successively as these methods are based on destructive sampling [12]–[14]. Today, the minirhizotron is the only nondestructive, *in-situ* sampling method for quantification of fine-root turnover at fine temporal scales.

The first rhizotron was a large, underground room constructed with glass walls allowing for observations of root growth and decay [15]. Cost and area of disturbance in the installation of these large rhizotrons was prohibitive, resulting in a modified approach: the minirhizotron, *i.e.*, buried cameras installed within transparent tubes that emerged in the 1980s [16]. Minirhizotrons are more cost effective, cause less disturbance, and allow for the analyses of successive root images to assess fine-root turnover through time [16], [17], from many species across multiple ecosystems [2], [8], [18].

Like all sampling methods, uncertainties in the minirhizotron approach have been identified. For example, Hendrick and Pregitzer [19] indicated fine-root production and mortality can be underestimated due to the time between sampling (*i.e.*, imaging of fine-roots) intervals. Villordon *et al.* [20] noted that a scanner-based minirhizotron grossly underestimated adventitious root quantity when compared to root quantity as measured by other methods. The limitations of minirhizotron cameras may also hinder accurate and precise estimates of fine-root stocks, turnover and biomass [21]. And even though measurement limitations such as these and others exist (*e.g.*, uncertainties in automated image analysis), minirhizotrons represent a robust and practical approach for quantification of fine-root turnover [22]. Here, we argue that the performance of minirhizotron cameras must be quantified in a reproducible and traceable manner to best quantify fine-root standing stock and turnover, and the processes they inform.

Some minirhizotron camera performances have been previously assessed [22], [23], yet a direct comparison of performance between different commercially available minirhizotrons has not been made. Standardized calibration methods and ‘best community practices' for quantifying measurement uncertainties of many environmental sensors (*e.g.*, resistance thermometers, pyranometers, etc.) exist, but such is not the case for minirhizotron cameras and their digital imagery. The absence of establishing such criteria from both the scientific community as well as standards-holding bodies hinder advances in design, deployment and data collection of minirhizotrons. The objectives of this paper are to present a reproducible, statistically traceable and defensible, steady-state approach for quantifying uncertainty and comparing key characteristics of minirhizotron camera performance (*i.e.*, digital imagery). We present measurement uncertainties for an array of performance metrics for two minirhizotron cameras. We also test for sources of human error and the potential for biases due to heating of the soil environment.

## Materials and Methods

### Selected Minirhizotrons

To our knowledge only three companies commercially produced minirhizotrons at the time of our study; i) Bartz Technology Corporation, Carpinteria, CA, USA, ii) CID-BioScience Inc., Camas, WA, USA, and iii) RhizoSystems LLC, Idyllwild, CA, USA. Minirhizotrons from two of the companies: the CI-600 In-Situ Root Imager (CID-BioScience), and the Automated Mini-rhizotron version A (AMR-A; RhizoSystems LLC) were used in developing a steady-state, laboratory approach to compare key functions of minirhizotron cameras and to quantify associated uncertainties. The AMR-A and CI-600 employ fundamentally different but common technologies for root imaging. The AMR-A is a fully automated, programmable instrument that uses a digital microscopic camera, and operates in a closed, sealed system [24]. The CI-600 is a manually controlled minirhizotron that relies upon scanner-based digital technology, and operates as an open system [25]. Choosing to analyze the performance of two different minirhizotrons also reflects the current range (albeit two) of technologies used.

### Quantification of Measurement Uncertainty

We assessed four key functions of minirhizotron cameras and propose methods to quantify and reduce associated uncertainties. We follow a traceable and statistically defensible approach (presented below) to quantify measurement uncertainty as proposed by the Joint Committee for Guides in Metrology [26]. Definitions of metrological terms used here are also consistent with the Standards-holding body (Table 1). The first step in calculating a measurement's uncertainty is to identify all input quantities, 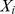, on which 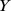 (the measurand) depends:
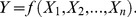
(1)


**Table 1:** Metrology terms and definitions.

Variable	Definition (is the-)
Accuracy	closeness of agreement between a measured quantity value and a
	true quantity value of a measurand; the concept ‘measurement
	Accuracy' is not a quantity and is not given a numerical quantity
	Value
Precision	closeness of agreement between indications or measured quantity
	values obtained by replicate measurements on the same or similar
	objects under specified conditions; the ‘specified conditions’ can
	be, for example, repeatability conditions of measurement etc.
Relative uncertainty	standard measurement uncertainty divided by the absolute value of
	the measured quantity value
Repeatability condition	condition of measurement, out of a set of conditions that includes
	the same measurement procedure, same operators, same
	measuring system, same operating conditions and same location,
	and replicate measurements on the same or similar objects over a
	short period of time
Reproducibility condition	condition of measurement, out of a set of conditions that includes
	different locations, operators, measurement systems, and
	replicate measurements on the same or similar objects
Resolution	smallest change in a quantity being measured that causes a
	perceptible change in the corresponding indication
Resolvable limit	minimum distance at which neighboring objects of the same
	intensity are resolved as two objects [28], [29]
Sensitivity	quotient of the change in an indication of a measuring system and
	the corresponding change in a value of a quantity being measured
Standard uncertainty	measurement uncertainty expressed as a standard deviation
Trueness	closeness of agreement between the average of an infinite number
	of replicate measured quantity values and a reference quantity
	Value
Type A evaluation	evaluation of a component of measurement uncertainty by a
	statistical analysis of measured quantity values obtained under
	defined measurement conditions, *e.g.*, repeatability condition
Type B evaluation	evaluation of a component of measurement uncertainty determined
	by means other than a Type A evaluation

All definitions are provided by JGCM [27] standards-holding body unless otherwise specified.

Because the measurement uncertainty of a measurand is a function of the uncertainty of its input quantities, the function 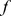 should contain every known, not necessarily quantifiable, source of measurement uncertainty, including all corrections and correction factors, unit conversions, etc. The standard uncertainty of each input quantity is quantified by two different uncertainty evaluation “Types”. Type A evaluation of uncertainty is based on first-hand statistical analyses and quantifies the distribution of 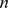 independent observations taken under controlled, often steady-state, conditions. Type B evaluation assumes *a priori* distribution for data and is not based on first-hand statistical analyses. In many cases, Type B uncertainties are taken directly from calibration specifications, typically provided by a manufacturer, or are arrived at using best scientific judgment. Here, we give a mathematical overview of a typical Type A evaluation. First, the mean of 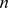 independent observations is calculated:
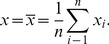
(2)


Second, the experimental standard deviation is calculated:
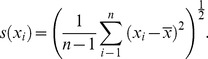
(3)


The standard uncertainty of the input quantity 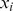 can either be the experimental standard deviation of the sample if quantifying the expected variance of an individual observation *or* the standard deviation of the mean (standard error) if quantifying the expected variance of a mean, such that,
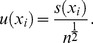
(4)


Each individual source of uncertainty (represented here by the standard deviation of the mean) then propagates to a final, combined uncertainty,
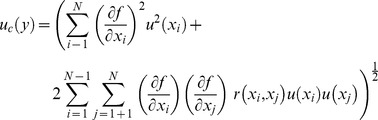
(5)where, 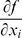 is the partial derivative of the function, 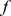, with respect to the input quantity, 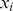; 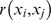 is the correlation coefficient computed by dividing the covariance, 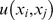, of the respective input terms by the product of their individual uncertainties,
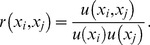
(6)


When input quantities are *uncorrelated*, the second term of Eq. (5) can be dismissed. However, for the case where the input quantities share a correlation of +1, Eq. (5) decreases to the linear sum of terms,
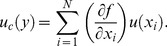
(7)


It is common practice to expand the combined uncertainty to a larger confidence level, usually 95%. To do so, the function (Eq. (5)) is estimated by a normal distribution with an effective degrees of freedom, 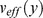, attained by the Welch-Satterthwaite formula,
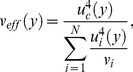
(8)where, 
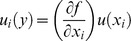
 represents the individual, partial uncertainties of the combined uncertainty, and 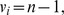 represents the degrees of freedom of each input variable. The expanded uncertainty at 95% confidence, 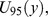 is then obtained by,
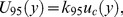
(9)where, 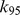 is the coverage factor (found in Table G.2 of [26]). It should be noted that deriving an expanded uncertainty using the Welch-Satterthwaite formula assumes input quantities are uncorrelated and independent. While this assumption typically holds true when measuring *in-situ* phenomena, it is widely applied for steady-state calibrations using repeated measures which further assumes the conditions of instrument uncertainty under *in-situ* and steady-state conditions remain the same. Currently, there is no metrological or statistical alternative to using the Welch-Satterthwaite formula that is accepted by the standards-holding body, and addressing this issue is an active area of research [30], [31]. The reader should also note that unless otherwise specified, all uncertainties presented in this manuscript are given in expanded form at 95% confidence. Data and uncertainty analyses for all procedures described in this manuscript are provided in Data S1.

### Assessing the Spatial Uncertainty of Camera Positioning

Repeated images of the same area provide a measure of how well *the same* root (stocks) and their turnover (a time delta) can be estimated [32]. In all minirhizotrons, the camera moves within an access tube to image the belowground environment. Hence, the ability to assess the spatial variability or precision of a moving camera to repeatedly image the same area is potentially a large source of uncertainty. In some cases, researchers have engraved marks onto minirhizotron tubes or utilized automatic indexing post-manufacture to quantify and possibly account for spatial uncertainties of camera location between sequential sampling times [22]. The minirhizotron tubes of the AMR-A and CI-600, however, did not include reference marks. The AMR-A camera has the ability to ‘auto-index’ image locations [24], and once the AMR-A completes a full pre-programmed scan of the tube the camera returns to a ‘*home*’ position located at the top of the minirhizotron tube. One method to assess the spatial variance would be to examine the AMR-A camera's auto indexed position for subsequent scans, however, at the time of this study, RhizoSystems LLC's Root View software did not record or archive such information. Because it is a manual minirhizotron, auto indexing was not available in the CI-600.

Here, we quantify the spatial uncertainty among a set of sequential images from each instrument. A stationary target (Kodak TL-5003 imaging test chart, Eastman Kodak Co., Rochester, NY, USA) was secured to an arbitrary location on the outside of each minirhizotron tube. The target was scanned thirty times (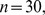 noted throughout the remainder of the text as “test”) at manufacturer-specified default settings (Table 2). In total, five tests were completed independently, three with the AMR-A (tests A1, A2, and A3), and two with the CI-600 (tests C1 and C2; Table 3).

**Table 2:** Specifications and manufacturer recommended default settings of each minirhizotron

Specification/Settings	CI-600	AMR-A
Tube length (cm)	182.0 (Others available)	156.8 (including handle and fin)
Tube diameter (cm)	6.4	10.8
Tube circumference (cm)	20.1	33.9
Operating range (°C)	Dependent on laptop	−12 to 45
Power requirements	Powered by laptop	5A @ 120 VAC
Removable camera	Yes	No
Operations	On site; field technician	Remotely; automated
Image dimensions (cm)	21.6×19.6	0.226×0.301
aresolution (ìm)	42.3×42.3 (∼24 pixels (p) mm^−1^)	4.7×4.7 (∼212 p mm^−1^)
Pixels/image	2.356×10^7^	3.07×10^5^
Images/full-tube scan	8 (with 182.0 cm tube)	3.2928×10^4^
Software	CI-600 Root Scanner v.3.1.22	Root View v3.0.0.7
bDefault camera settings	Brightness = 0; (50%)	Camera Exposure = 8; (60%)
	Contrast = 0; (50%)	LED camera lights = 192; (100%)
	Threshold = 0; (50%)	Dwell time = 100 ms

a manufacturer recommended resolutions (B Meyer, CID-BioScience Inc., pers. comm.; M Taggart RhizoSystems, LLC, pers. comm.)

b settings provided with percentage relative to the maximum range of the particular setting [24], [25]

**Table 3:** Description of camera position tests.

Test	Condition of measurement	Description
A1	Reproducibility	AMR-A: Camera homed between subsequent scans;
		deviation from home location *was greater than* the
		manufacturer recommended 20 µm
A2	Reproducibility	AMR-A: Camera homed between subsequent scans;
		deviation from home location *was equal to or less than*
		the manufacturer recommended 20 µm
A3	Repeatability	AMR-A: Camera not homed between subsequent scans
C1	Reproducibility	CI-600: Removal and replacement of camera
C2	Repeatability	CI-600: No removal and replacement of camera

Each test had 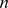 = 30.

To quantify the reproducibility of the AMR-A camera, two tests were conducted under conditions where the camera was programmed to return to its ‘*home*’ position, *i.e.*, the manufacturer's reference location, between subsequent scans of the target. For best results the manufacturer recommends the camera returns to within ±20 µm of its ‘*home*’ position among subsequent scans, thereby managing the reproducibility. The first test (A1) comprised scans where the radial (around the tube) and vertical (along the tube) distances between the camera's actual position and its ‘*home*’ position exceeded ±20 µm among subsequent scans. The second test (A2) comprised scans where the radial and vertical distances between the camera's actual position and its ‘*home*’ position were ≤±20 µm among subsequent scans.

To quantify the repeatability of the AMR-A's camera position, the A3 test was conducted such that the camera was preprogrammed to image a 1 cm^2^ area starting at predetermined x (radial) and y (vertical) coordinates within the tube. Between subsequent scans, the camera was not programmed to return to its ‘*home*’ position. This test was designed to assess the variability of the camera's location independent of sending the camera to its ‘*home*’ position among subsequent scans.

Under field conditions, the CI-600 is manually installed into its tube. Hence, the C1 test simulated field conditions where measurements would be prone to human error through the removal and replacement of the camera by the operator (*i.e*., reproducibility). This test consisted of i) aligning the CI-600's reference dots with a fixed location at the tube's inlet, as recommended by the manufacturer [25], ii) placing the CI-600 camera inside the minirhizotron tube, iii) pushing it to a predetermined depth of the tube (target location) where the camera “locked” into place via its attached placement rod (the camera was pushed to the same depth for all scans), iv) allowing the camera to complete a full, ∼360^°^ radial scan, and then rotate back to ∼0^°^ from its starting position (an automated process among subsequent scans [25]), and v) removing the CI-600 camera from the tube; this method was repeated thirty times. The C2 test followed the same method as C1 with the exception that thirty successive images were taken under identical and stable conditions without removing the CI-600 from the tube (*i.e.*, repeatability). Test C2 was intended to quantify the spatial uncertainty of the camera free of external factors such as the influence of human error introduced in estimating reproducibility via test C1.

Because the image size of each minirhizotron camera is different (Table 2), two different sized targets of the Kodak TL-5003 test chart, a crosshair and a line-segment, were imaged by the CI-600 and AMR-A cameras, respectively. This ensured that the entire target was captured within an individual image rather than a mosaic of images, thus eliminating another potential source of uncertainty.

The Interactive Data Language (IDL) v8.1 (Exelis Visual Information Solutions, Inc., Boulder, CO, USA) was used to assess each image in grayscale format, and using the *thresholding* and *contouring* functions [33], discern pixels that comprise the target of interest from background pixels. To do so, the pixel intensity (0 (black) to 255 (white)) threshold was initially set to 253 and the pixels that comprised intensities less than this threshold test were then outlined with the contour function. Contouring was used here to ensure that only *one* outline for the entire black colored target was identified, *i.e.*, pixels not part of the target were excluded, and that *only* the coordinates of the pixels that included the target were identified. We then tallied the number of contours that were returned. If more than one contour was identified, the threshold value was lowered by one and the process was repeated until only *one* contour was identified in each image, *i.e.*, dynamic thresholding. Once the final threshold was determined (in this case, it was lowered by a total of 3, thus determining the final threshold of 250), the x and y coordinates of each pixel comprising the target from all images (*i.e.*, those with intensities less than the final threshold value) were outputted to separate data files and labeled by the test and image number.

We then quantified the central position of the respective object's location in each image by calculating the mean x and y coordinates of those pixels passing the threshold test. This was completed using Perl (v5.10, www.perl.org). By computing the object's central location in sequential images (at time one, time two, time n), we were then able to determine the difference in this central location among images and hence, determine the ability of the sensor to repetitively sample the same point(s) in space. We present the spatial uncertainty of the camera via one standard deviation (SD), because in this case, we think it is more informative to do so in the context of the sample variance, rather than the variance of the sample mean, *i.e*., standard error. The standard deviation can then be used in part to compute the expected spatial uncertainty of the camera (for an individual scan) relative to the camera's “true” location.

### Digital Imaging Performance

#### Evaluating Camera Sensitivity

Accurately quantifying the size of fine-roots may be limited by camera sensitivity and pixelation inherent in digital imagery. The pixelation process averages the signals across a given area, which subsequently decreases the overall sensitivity of a given technology, and can cause large uncertainties when fine-roots are imaged with cameras comprising low resolution, *i.e*., large pixels [34]. To assess the cameras' abilities to quantify root diameter, we determined the sensitivity of each minirhizotron camera, quantified measurement trueness over a range of known diameters, and computed the precision of such measurements.

Individual, black line segment widths of a truncated fan pattern (Kodak TL-5003 imaging test chart) were measured at the thirteen designated points (Table 4) by a Model 1602 Filar Micrometer (Los Angeles Scientific Instrument Company, Los Angeles, CA, USA), traceable to National Institute of Standards and Technology's (NIST) standards (NIST Test# 821/253660-94) at Applied Image Inc. (Rochester, NY, USA). These measurements were used to calibrate the minirhizotron measurements.

**Table 4:** Line widths and expanded uncertainties as measured by the NIST traceable micrometer.

Line Width (µm)	±U_95_ (µm)
409.63	2.22
348.21	1.64
303.53	1.64
268.48	1.64
242.62	1.64
194.06	1.64
161.95	1.38
120.27	1.38
100.15	1.38
73.81	1.38
57.99	1.38
43.66	1.38
39.29	1.38

Each minirhizotron camera imaged the truncated fan pattern under steady-state conditions (*i.e.*, no camera removal) and operated with the highest manufacturer recommended resolution. For the AMR-A this was ∼212 pixels (p) mm^−1^ with pixel sizes of 4.7×4.7 µm [24], and for the CI-600, this was ∼24 p mm^−1^ with pixel sizes of 42.3×42.3 µm [25]. Each minirhizotron camera operated inside a larger opaque tube to mimic underground conditions and eliminate exposure to extraneous sources of light. The resultant images were saved in bitmap (*.bmp) format to avoid data compression.

**Figure 1:**
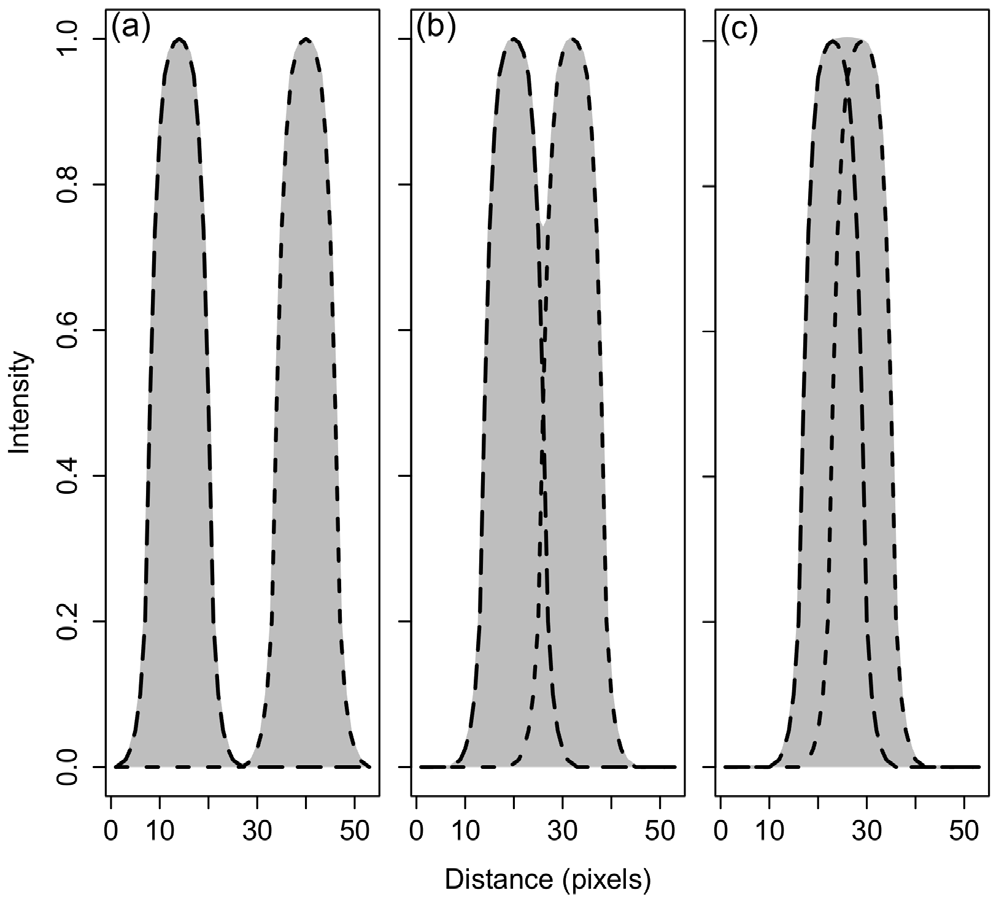
Conceptual diagram depicting the resolution of two objects (smooth contours) with equal intensities at varying distances. The dashed lines denote two conceptual objects of equal intensity, and the shaded, gray area represents what the camera resolves; (a) fully resolvable, (b) just resolvable (Rayleigh Criterion), and (c) unresolvable, *i.e.*, both objects are resolved as a single object.

We assessed the minirhizotron images using IDL. The intensities and coordinates of each pixel residing within the thirteen rows perpendicular to the truncated fan pattern (*i.e.*, those points measured by the NIST traceable micrometer) were outputted to a *.csv file. Data were then normalized with respect to the neighboring maxima. We followed the Rayleigh Criterion to quantify the resolvable limit (sensitivity) of each minirhizotron (Figure 1). This criterion can be thought of as defining the minimum distance between two objects that can be individually resolved by a camera [28], [29]. More specifically, we also define this minimum distance when a contrast of 26.4% exists between two neighboring maxima with identical magnitudes [35]. In the case of pixelated images, *magnitudes* relates to pixel intensities (signals). Pixel intensity graphs (Figure 1) were generated via Microsoft Excel 2010 (Microsoft Inc., Redmond, VA, USA) to assess sensitivity and quantify the Rayleigh Criterion.

#### Quantifying Root Diameter

Measurement trueness was quantified through a number of steps. First, pixel intensity data (outputted by IDL) of the six widest lines measured by the micrometer (Table 4) were ingested into a Perl script. These six points were chosen because they did not reside near the resolvable limit of either minirhizotron camera. Thus, we standardized the approach to make robust comparisons between both minirhizotron cameras. Second, we used two separate test methods to estimate trueness of line thickness; we tallied the number of successive pixels with intensities less than i) the threshold of 250 (as determined by the camera positioning tests), and ii) half of the maximum intensity of an individual group of successive, black pixels. The latter follows the Full Width at Half Maximum (FWHM) approach, a common method used in optics [28]. The maximum intensity value for FWHM was independently calculated for each group of successive, black pixels. Successive, black pixels from each test were tallied separately, thus quantifying the width of a black line-segment in two different manners. Third, the mean line segment widths of all the black line segments at each of the six points were calculated in units of microns following Eq. (2). Fourth, the standard errors of these six mean line thicknesses were calculated following Eq. (3) and (4). Mean data from each of the minirhizotrons were then fit to a calibration curve against those values quantified by the micrometer.

Uncertainty estimates for uncorrected and corrected (*i.e*., those data not fit and fit to the calibration curve, respectively) data were estimated and compared. In both cases, input data were considered uncorrelated and independent. Combined uncertainties were derived following Eq. (5). Sources of quantifiable uncertainties of each minirhizotron dataset included: i) uncertainty of the micrometer measurements (Type B uncertainties taken from calibration specifications), ii) measurement trueness (uncorrected or corrected), and iii) the precision of the minirhizotron measurements. The average of the computed standard errors over the entire measurement range was used to calculate the precision of each camera's ability to quantify root diameter. Individual uncertainties were summed in quadrature following Eq. (5), and resulting combined uncertainties were then expanded following Eq. (8) and (9).

#### Assumptions

It should be noted that two assumptions were made when determining the resolvable limit of each camera and their ability to quantify root diameter. First, we assumed each camera operated using a constant focal length, such that the focal point was always positioned at the outer edge of the respective minirhizotron tube, i.e., where the truncated fan pattern was secured, and not at some distance d (mm) away from the outer edge of the tube. Second, it was assumed that the measured widths of the line segments (two-dimensional objects) were representative of root diameters (three dimensional objects; see [23]). We cannot discount the possibility that these assumptions may lead to additional uncertainties. Regardless, the presented approach is reproducible, statistically traceable, and serves as a guideline for future researchers to build upon.

#### Quantifying Root Color

Overestimation of fine-root lifespan and subsequent underestimation of fine-root turnover are possible if fine-roots are only classified as dead once they disappear from the view of the minirhizotron camera [10]. To address this problem, some researchers quantify the age and/or health of fine-roots as a function of color. However, the color nomenclature used by researchers to define root health is unstandardized and ambiguous. For example, the terms *white* [10], [36]–[38] and/or *cream* [32] are used to designate living and/or new roots, *brown* [32], [36]–[38], or *woody* [32] to describe dying/aging roots, *dark* to describe older roots [10], and *dark brown*, *very dark brown* [39] or *black* to describe dead roots [32], [37], [39]. Interestingly, the term *brown* is used to describe the colors *orange* and/or *dark yellow* imaged at low luminance [40]. In a recent paper, Dannoura *et al.* [36] quantified root-color changes via digital imagery to discern between living and dying/dead roots, but did not provide an estimate of the imager's ability to quantify color with a known trueness and precision. The color space vectors outputted by digital cameras, *e.g.*, RGB, are device-dependent, do not usually adhere to the fundamentals of Colorimetry [41], and the spectral response to color among a population of cameras can vary greatly [42]. In other words, the same root stock may appear *brown* when imaged by camera A, *cream* colored when imaged by camera B, and *dark brown* when imaged by camera C. “True” root colors cannot be known and comparisons cannot be made using device-dependent imagery (and qualitative color terms). To our knowledge no researchers have identified device-independent color space, *e.g*., CIE XYZ, CIE L*a*b*, etc., thresholds for digital imagery to discern among new/living, dying/aging, or dead roots, nor have they provided color space uncertainty estimates.

JCGM [26] notes that any measurement is not complete without a supplementary uncertainty estimate; this is especially true for color measurements, as many factors, such as the lighting and the geometry of measurement, can influence the uncertainty of its measurement [43]. Here, we present an uncertainty analysis of color quantification of the two minirhizotron cameras and also stress the importance of monitoring root age and/or health in a quantitative manner via a device-independent color space.

In many cases, spectral reflectance of a sample is quantified by concurrently measuring signals of a sample and a standard under identical measurement conditions [44]. For this study, however, spectral reflectance of the samples and the standards were quantified independently, as this is becoming a more common approach (Gary Reif, Applied Image Inc., Personal Comm. 2014). A Color Test Chart (QA-69-P-RM, Applied Image Inc.) containing eight color patches was measured by a NIST traceable X-Rite eXact densitometer (X-Rite Inc., Grand Rapids, MI, USA) at Applied Image Inc. This densitometer directly measured reflectance of the sample over a spectrum of 400 to 700 nm 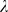 at 10 nm increments while operating with International Commission on Illumination (CIE) [45] Illuminant D50, 0/45° geometry of measurement, and the CIE 1931 2° standard observer. This was completed five separate times for each of the color patches to inform uncertainty of the reflectance measurements. In total, five datasets of 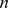 = 31 (reflectance data from 400–700 nm 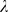 at 10 nm increments) *for each* of the eight colors were generated. The five datasets of reflectance data for each color patch were then averaged together at each wavelength. Although the CIE [45] recommend that data should be captured at or interpolated to 5 or 1 nm increments to avoid uncertainties due to gaps, research shows that the increase of uncertainty in reflectance data when capturing data at 10 nm increments is likely trivial compared to data captured at 5 or 1 nm [46]. Moreover, interpolating to these increments can actually lead to larger uncertainties (than the 10 nm data) depending on the interpolation scheme (*e.g.*, linear, Lagrange). Therefore, we chose not to interpolate the reflectance data. Following recommendations from the CIE [45] and the American Society for Testing and Materials (ASTM) [47], reflectance data were then converted to the CIE 1931 XYZ color space with the following equations,
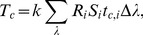
(10)where, 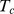 represents the tristiumulus values: 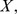 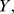 and 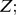 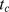 represents the color matching functions: 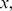 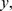 and 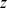 associated with the appropriate tristiumulus value, 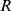 represents reflectance, 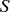 is the relative spectral power distribution of the illuminant, 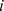 is the individual wavelength, and 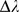 represents the wavelength interval. The variable 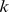 is a normalizing constant defined by:
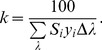
(11)


Resulting tristiumulus values were then converted to x and y chromaticity coordinates via:
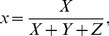
(12)and
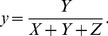
(13)


Tristiumulus values and resulting chromaticity coordinates were then used to characterize (calibrate) the minirhizotron data.

Each minirhizotron was characterized independently. The Color Scanner Test Chart was secured to the outside of the respective minirhizotron tube, and the minirhizotron was placed inside of a larger opaque tube to eliminate ambient light. The Color Scanner Test Chart was imaged once while each minirhizotron operated at manufacturer-specified default settings (Table 2). The images captured at default settings were then analyzed for over-saturation (over-exposure). If the colors imaged by the minirhizotron were over-exposed, we adjusted the camera settings and reimaged the chart until the influence of saturation was mitigated. We utilized bitmap (*.bmp) format for all the images to avoid data compression. After the optimal camera settings were derived, the Color Scanner Test Chart was imaged five separate times by each minirhizotron camera. Images were uploaded to Adobe Photoshop (Adobe Systems, San Jose, California, USA) to inform the respective device-dependent color space used by each camera. The digital color space of images from both minirhizotrons could not be verified. As such, the individual vector intensities of each of the eight colors imaged by the two minirhizotrons were assumed to be standard RGB (sRGB), and were analyzed using IDL. Pixels residing along the boundaries of each color patch were discarded from the analysis to avoid inadvertent quantification of merged colors. Standard RGB data from each minirhizotron were then characterized (calibrated) to the CIE XYZ data of the densitometer following a linear transformation [48]:
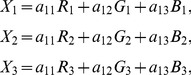
(14)where, 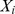 is a tristiumulus value of an individual color patch (converted from spectral reflectance data measured by the densitometer), 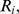 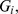 and 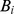 are the values outputted by the minirhizotron camera for the same color patch, and 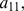 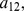 and 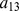 are empirical coefficients. The same calculations were carried out for 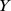 and 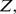 for a total of nine equations. Three colors: *white*, *amber* (a mixture of *orange* and *yellow*), and *yellow*, were used for the linear transformation (Table 5). These colors were chosen as they best represent the region of expected root colors relative to the other five colors of the test chart. The resultant tristiumulus values were then converted to chromatic coordinates following Eq. (12) and (13).

**Table 5:** Mean chromaticity coordinates (and expanded uncertainties) converted from reflectance data measured by the densitometer.

Color	x	±U_95_(x)	y	±U_95_(y)
Yellow	0.463	0.001	0.494	0.001
Amber	0.424	0.001	0.397	0.001
White	0.349	0.001	0.363	0.002

Color space characterization, especially for applications relying on digital imagery (RGB), is an active area of research. Various techniques to characterize RGB to a CIE color space have been investigated [48]–[50]. As cautioned by these studies, the use of a linear calibration using only three colors will cause large uncertainties when attempting to characterize other colors via the derived coefficients 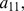 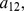 and 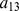. Here, we acknowledge this issue; however, one goal of this study was to develop a method of traceable, standard, and defensible means to estimate the uncertainties in minirhizotron imagery. The extent of our characterization was limited by the number of colors on our test chart, and thus a characterization that includes the many possible combinations of tristiumulus values and higher order polynomial modeling was beyond the scope of this investigation.

An uncertainty analysis for color is estimated following the JCGM [26], Early and Nadal [44], the CIE [45], and Sándor *et al.* [46]. Uncertainty estimates of the spectral reflectance data (as measured by the densitometer) were provided as relative values by Applied Image Inc. and are considered Type B uncertainties. Because the spectral reflectance data are the rawest form of data obtained from the densitometer, we assumed that the corresponding uncertainty values provided by Applied Image Inc. encompassed any correlations among the signals of the standard and the sample, and are corrected for any systematic uncertainties inherent in reflectance data (as mentioned in [44]). Correlations among wavelengths and among tristiumulus values were quantified following Eq. (5), and the equations in [44]. Combined uncertainties of each tristiumulus value, 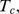 and each chromaticity coordinate, 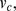 respectively, were derived via,
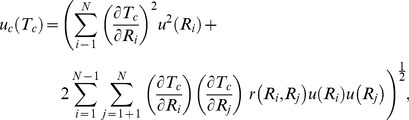
(15)and,
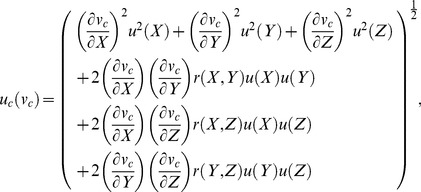
(16)


We present uncertainty estimates of uncharacterized (*i.e*., sRGB vectors), and characterized (*i.e*., 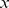 and 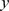 chromaticity coordinates) in expanded form. The act of characterizing from sRGB to the chromatic coordinates is a calibration procedure, and thus, we cannot display uncharacterized minirhizotron data in the form of 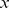 and 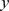 chromatic coordinates.

### Estimating Heat Dissipation

It is well known that temperature controls many enzymatically-driven metabolic processes in the soils [51]–[53]. Artificial changes in soil temperature could, in turn, change plant and microbial responses. For example, a meta-analysis by Rustad *et al.* [54] concluded that nearly all soil warming experiments increased soil respiration, N mineralization, and plant productivity. The AMR-A operates as a closed system and uses the ground as a heat sink [24], which may impact fine-root turnover. To assess the potential impact of heat additions to the soil environment, we measured the soil temperatures surrounding the installation of the AMR-A. We did not measure the soil temperatures around the CI-600 as it is not a closed system and we assumed its scanner-based imager dissipated a negligible amount of heat to the surrounding environment during the time it takes the CI-600 to complete a full-tube scan (*i.e.*, roughly thirty-minutes).

**Figure 2:**
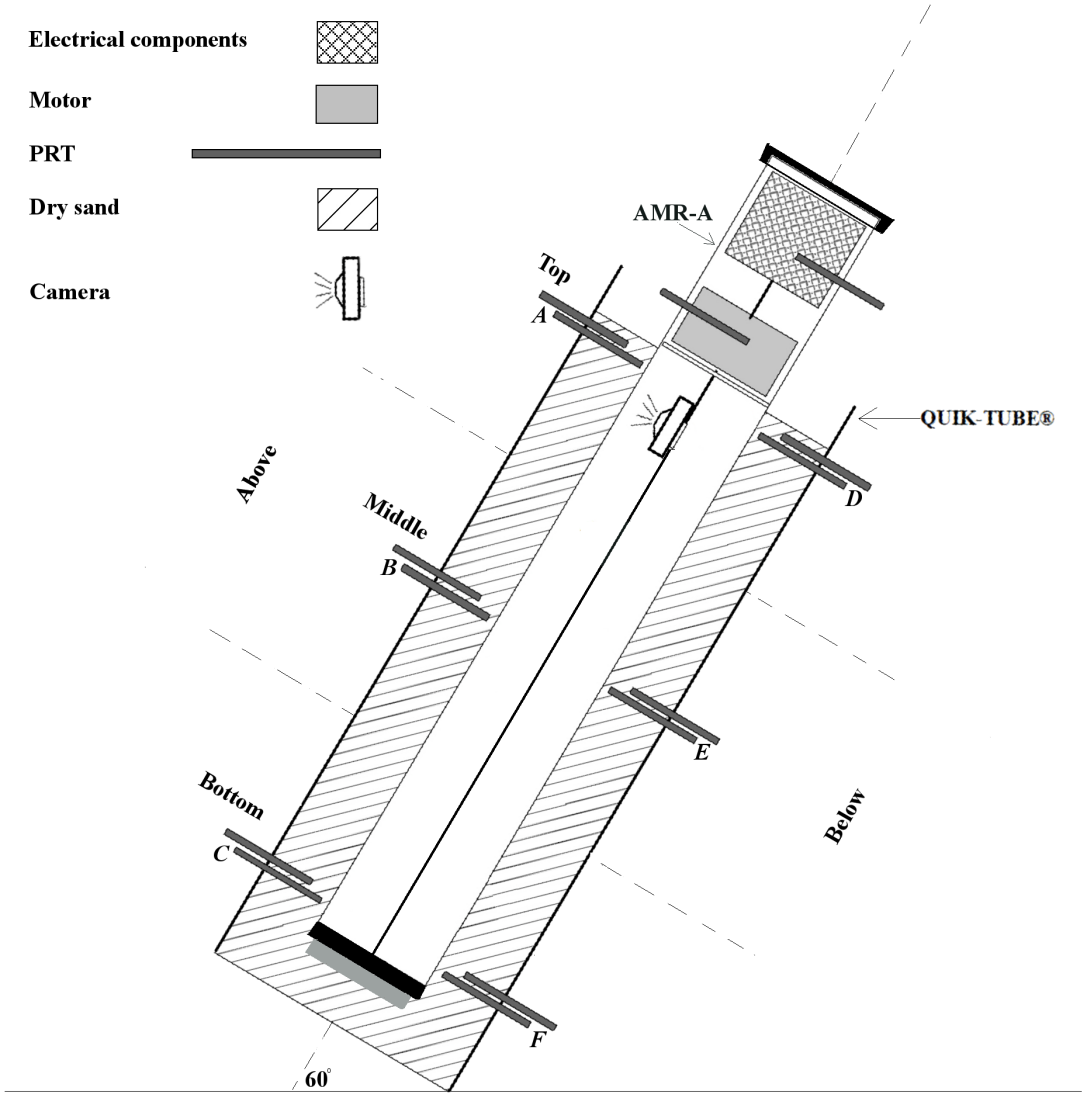
Experimental design to determine heat dissipation. A cross-section schematic of the experimental design used to assess heat dissipation (not to scale) for the AMR-A. The three depths: *Top*, *Middle*, and *Bottom*, correspond to depths of 5, 48, and 86 cm below the sand surface, respectively. The locations of the PRTs residing 0.5 cm from the minirhizotron are denoted by letters *A* through *F*, and correspond to the results shown in Figure 5. The LED lights reside on the body of the camera. The 60° angle refers to the overall installation relative to the horizontal (plane).

The AMR-A was placed into a 118 cm (L) x 30 cm (ID) QUIK-TUBE (part number 6922, QUIKRETE, Atlanta, GA, USA) and the area between the exterior of AMR-A's tube and interior of the QUIK-TUBE was filled with dry, QUIKRETE, All-Purpose sand (part number 1152). Dry sand was chosen because of its uniformity and low specific heat capacity, thus ensuring that our approach quantified a “worst case” scenario with regard to temperature increase due to heat dissipation from the AMR-A. A pair of holes was drilled at three different levels, and on opposing sides of the QUIK-TUBE. The location of these holes coincided with the AMR-A camera's home position (top), mid-level of the AMR-A's tube (middle) and the lowest possible camera scanning level achieved by the AMR-A (bottom; Figure 2). One four-Wire Platinum Resistance Thermometer (100 Ω PRT; Thermometrics Inc., Northridge, CA, USA) was inserted into each hole, thus allowing each PRT to reside perpendicular to the AMR-A's tube. At each level, the tip of one PRT was positioned 0.5 cm away from the edge of the AMR-A tube. This suite of 6 PRTs remained fixed for this entire heat dissipation test (*A* through *F* in Figure 2). We then positioned another suite of 6 PRTs at the same locations and in the same manner, but inserted them at constant distances of 7.0, 3.0, and 2.0 cm from the AMR-A's tube during three sequential tests, respectively. We then mounted two PRTs 0.5 cm from the AMR-A's tube at heights of 10 and 30 cm above the surface of the sand to measure temperature changes of ambient air near the AMR-A's electrical components and motor. Prior to testing, all PRTs were calibrated against NIST's standards (*i.e.*, the triple point of mercury and water, and melting point of gallium) and uncertainty analyses were generated following JCGM [26]. Data from these fourteen, calibrated, PRTs were acquired with a datalogger (CR3000, Campbell Scientific Inc., Logan, UT, USA) at 0.1 Hz. Before testing began the QUIK-TUBE and AMR-A were collectively tilted at an angle of 60^○^ from horizontal (plane) to mimic the angle minirhizotrons are usually installed in the field [22]. This allowed our setup to mimic the dynamics of heat flow similar to those encountered in the field.

Three, individual, full-tube scans were conducted over a ten-day period. During testing, the AMR-A operated with manufacturer-specified, default camera settings (Table 2). Upon completion of each full-tube scan, the camera of the AMR-A was programmed to return to its home position and was powered down. We allowed for the thermal mass of the sand/tube assembly to equilibrate with ambient conditions for a minimum of 48 hours between each test scans. Mean, ambient lab temperature was 23.21±0.21°C (mean ± 1 SD).

Temperature data were split into three different groups, one for each test. Temperature change at each location was quantified relative to the temperature of the respective PRT at the start time of each full-tube scan.

## Results and Discussion

### A Caveat

Because we did not test a population of minirhizotron cameras, we cannot rule out that our findings are a function of camera-specific characteristics. We did, however, receive assurances from the manufacturers that the tested cameras were representative of a larger population. Two separate CI-600 In-Situ Root Imagers were used during testing. Both experienced mechanical failures that required replacement or manufacturer repair.

### Spatial Uncertainties of Camera Positioning

Both the repeatability and reproducibility of the AMR-A camera's position were significantly better than that of the CI- 600 (Table 6). Regarding reproducibility, the radial and vertical uncertainties of the CI-600 camera (test C1) were *two to three orders of magnitude larger* than the radial and vertical uncertainties of the AMR-A's camera (*i.e.*, A1, and A2). The larger spatial uncertainty of the CI-600's camera position for test C1 is most likely a result of the extraction and replacement of the camera between subsequent scans, a task that mimics how the CI-600 is actually used in the field. The discrepancies of spatial uncertainties for tests A1 and A2 are most likely due to the camera's ability to consistently return to the home position between subsequent scans. In regards to repeatability, the differences between the spatial camera uncertainty of tests A3 and C2 were not as large as those of the reproducibility tests. However, the uncertainties quantified during test C2 were still larger in the radial and vertical dimensions when compared to test A3 (see Table 6). The differences in spatial uncertainties between these two cameras were most likely a result of the operational characteristics of the minirhizotrons, *i.e.*, automated vs. manual camera positioning. That said, we also interpret the large difference between the spatial uncertainties of camera location during tests C1 and C2, is due to the CI-600's removal and replacement from the tube. In other words, the influence of human sources of error is evident in test C1, and the potential magnitude of this error can be quite large. When human interaction was completely removed from testing (*i.e.*, C2) results improved (Table 6).

**Table 6:** Precision of minirhizotron camera location for each sampling method; values are provided as *one standard deviation*.

	Precision	
Test	Radial (degrees)	Vertical (µm)
A1	0.12	24.87
A2	0.01	4.32
A3	0.01	38.37
C1	13.72	1942.23
C2	0.22	84.06

Human operation and interaction with the technology is an essential aspect of the CI-600 performance. The CI-600 camera needs to be physically moved among field-deployed tubes and moved to different depths of a tube during sampling. Although such an approach can be beneficial in terms of cost, it leads to additional uncertainties. In working with the C1-600, precautions were taken to minimize misalignments of the camera with respect to a reference point at the tube's inlet, as recommended by CID-BioSience Inc. [25]. We assume that our carefully controlled laboratory conditions would result in performance superior to those obtained under field conditions.

An underlying goal was to provide a traceable method to assure the same area of roots is imaged across successive sampling intervals. One solution is creating reference points along the tube via indexed marks or etchings [32], [55]. Given the large spatial uncertainties in the CI-600 camera found in this study (Table 6), the CI-600 is currently incapable of imaging the same area of root stocks at sequential sampling intervals without the use of etchings or reference points along the tube. Providing such indexed etchings to all minirhizotron tubes, including the AMR-A, provides the ability to assess the instrument's spatial uncertainty *a posteri*, as well as manage any changes that occur over time, *e.g.*, mechanical wear. Etchings on the tube would also aid in the alignment of neighboring images when producing a larger ensemble of images, however, estimating the uncertainties of combining neighboring images into a larger mosaic were outside the scope of this study.

### Digital Imaging Performance

#### Camera Sensitivity

The AMR-A was capable of resolving smaller objects than the CI-600. The CI-600 was able to resolve line widths at 100.15±1.38 µm, but was unable to resolve line widths at 73.81±1.38 µm. Since the minirhizotron cameras were calibrated at points, we cannot state (empirically) the exact location where the CI-600 reached its resolvable limit, however, we empirically bound its resolvable limit between 72.43 and 101.53 µm. Vamerali *et al.* [56] found that at least two pixels are needed to resolve the diameter of an object. Thus, it is reasonable to conclude that the resolvable limit of the CI-600's camera is 84.6 µm, *i.e.*, the width of two of its pixels. The ARM-A camera's resolvable limit was unable to be empirically quantified because the camera was able to resolve the narrowest line widths, 39.29±1.38 µm, of the truncated fan pattern. Provided the same reasoning as above, we conclude that the resolvable limit of the AMR-A is 9.4 µm.

Although fine-root diameters can be resolved with two pixels, this does not guarantee that fine-roots of 84.6 µm (CI-600), and 9.4 µm (AMR-A) will be resolvable in the field with varying environmental conditions, root orientations, and potential camera photocell degradation. This is also true if the object is not aligned with the pixels themselves. Zobel [23] states that, ideally, pixel size should be 25% of the diameter of the smallest root being imaged. When we follow Zobel's [23] research and incorporate our findings, the conservative resolvable limits of the CI-600 and AMR-A for operational use in the field would be 169.2 µm and 18.8 µm, respectively (with the given instrument settings used in this study, Table 2). As noted previously, Villordon *et al*. [20], underestimated root quantity using a scanner based minirhizotron (*i.e*., the CI-600). Given the CI-600 settings that the researchers utilized (*i.e.*, 78 dots (d) cm^−1^ or ∼8 p mm^−1^, with pixel sizes of 127×127 µm), the resolvable limit using the Rayleigh Criterion would be 254 µm. Applying Zobel's [23] findings (above) to the research of Villordon *et al*. [20] would result in fine-roots with diameters <508 µm to go virtually undetected; this may provide insight as to why root quantity was underestimated, and stresses the importance of knowing the limits of digital imagery prior to field use.

#### Neighboring Objects of Different Sizes

Mean, fine-root diameters have been estimated globally as 220, 440, and 580 µm for grasses, shrubs, and trees, respectively [57], while the range of fine-root diameters are often defined as 0–3000 µm [8], [34], [58], [59], and fungal hyphae diameters range from ∼2–80 µm [60]. Given that fine-root diameters can be quite small, argues that the resolvable limit should be reported for such studies. This is the first study to report a defensible and traceable means to determine the resolvable limit of current, commercially available, minirhizotron cameras.

Interestingly, we found a change in the cameras' responses to contrast. The signals of the black line segments became progressively more saturated as the test lines became narrowed. In other words, the signals of the black line segments became larger, *i.e*., moving away from the lower end of the 0–255 intensity range, and the contrast between the black and white line segments decreased. At a line width of 409.63±2.22 µm the mean pixel intensities were 133.02±2.39 and 121.51±1.02, as imaged by the CI-600 and AMR-A, respectively. At a line width of 194.06±1.64 µm the mean pixel intensities were 146.20±3.53 and 139.25±1.41, as imaged by the CI-600 and AMR-A. We interpret the change in the cameras' abilities to consistently detect contrast to i) the backscattering of light caused by the increased area of white background surrounding the truncated fan pattern as it narrowed, and the ii) signal averaging inherent in the pixelation process. It may also be a result of the analysis software's ability (in this case, IDL) to convert color images to grayscale. Our finding poses a challenge, as it suggests that the two cameras have difficultly resolving neighboring objects of different sizes, *i.e.*, they may have difficulty resolving and quantifying neighboring fine-roots with different colors or brightness, or when there is a range of root diameters.

#### Root Diameter Measurements

**Figure 3:**
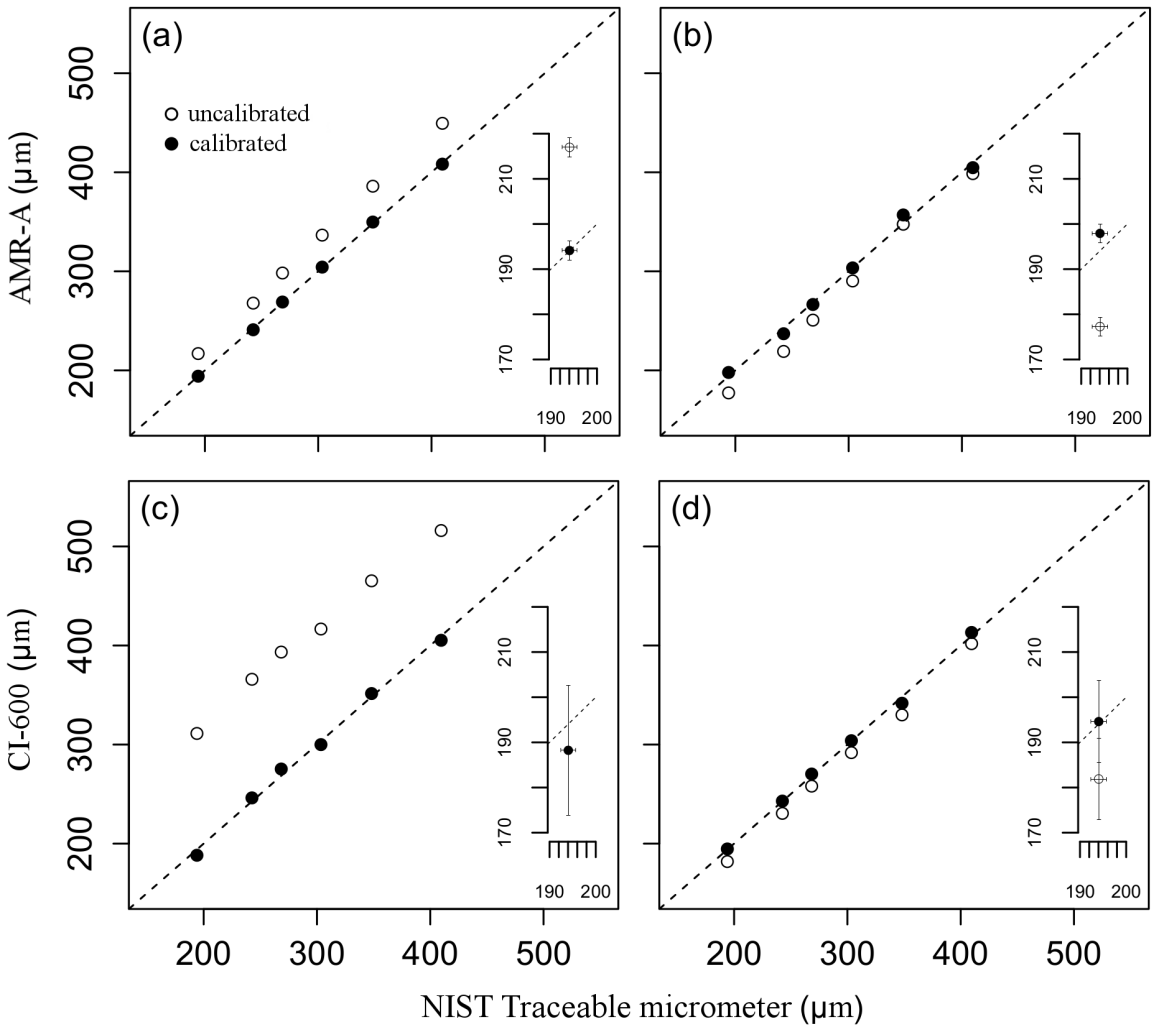
Line widths as measured by both minirhizotrons. Estimated line widths as determined by both minirhizotrons cameras against those of the NIST traceable micrometer. Results of the AMR-A are shown in (a) using the 250 pixel intensity threshold (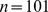), and (b) using the FWHM threshold (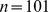). Results of the CI-600 are shown in (c) using the 250 pixel intensity threshold (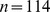), and (d) using the FWHM threshold (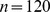). A 1∶1 line is shown on each graph. Inset plots are provided at the 194.06±1.64 µm calibration point to emphasize the magnitude of random uncertainty and show that calibration can correct for bias, but not random uncertainty. Error bars are given as ±2 SE for uncalibrated data and ±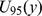 for calibrated data.

Prior to calibration both minirhizotrons exhibited measurement bias when quantifying root diameter. Using the 250 threshold method, the AMR-A overestimated (*i.e.*, a positive bias) root diameter by an average of 32.03±3.06 µm (Figure 3a), while the CI-600 overestimated by an average of 117.16±13.97 µm (Figure 3c). Using the FWHM method, the AMR-A underestimated (*i.e.*, a negative bias) root diameter by an average of 15.51±12.50 µm (Figure 3b), while the CI-600 underestimated, on average, by 12.47±7.68 µm (Figure 3d). At first glance it may seem that using the FWHM method yielded better results because the absolute bias is smaller than that of the 250 method. Interestingly, however, using the FWHM method, the AMR-A camera grossly underestimated approximately 6% (*i.e.*, 6 of 101) of the samples and skewed the dataset; the result of which was reflected not only in the average systematic bias of -15.51 µm, but also in the large standard error of ±12.50 µm. However, these same 6 samples (line widths) were not significantly different than the other 95 samples when using the signal threshold of 250. The results of the FWHM test suggest a skewed distribution and a potential systematic bias if the products are used for turnover and below ground productivity estimates. If the degree of systematic bias is constant, then the delta calculation of root growth and/or senescence between sequential images in time will be small. If the bias is not constant, the contributing uncertainties will be difficult to quantify over time.

The ability of each minirhizotron camera to quantify root diameter was greatly improved after calibration. The systematic biases of both cameras were corrected and resultant expanded uncertainties for both minirhizotron cameras using either analysis method reduced to within ±17.00 µm of the estimated “truth”. The largest fraction of the expanded uncertainty for the calibrated data resulted from the reproducibility of the cameras' abilities to quantify root diameter. Calibration can correct bias, but it cannot mitigate the estimate of uncertainty in the form of repeatability and/or reproducibility (see inset plots of Figure 3).

#### Root Color Measurements

When compared to one another, both minirhizotrons exhibited significant differences in quantifying the sRGB vector intensities of each of the eight colors (Table 7, Table 8). At first, we operated using the manufacturer-specified default settings, and three of the color patches: *magenta*, *yellow*, and *amber*, imaged by the AMR-A were over-exposed and all sRGB vectors were 255, *i.e.*, the highest possible pixel intensity. To mitigate over-saturation, the exposure of the camera was adjusted. We empirically determined that the AMR-A was capable of quantifying all eight colors (within reason) when operating at a lower camera exposure of 12, (*i.e*., not the recommended, default exposure; Table 7). Moreover, many of the AMR-A images were also prone to areas of overexposure in the top left corner of each image, regardless of which exposure was used. The CI-600 also exhibited signs of over-saturation while operating at default settings. Specifically, the R vectors of *magenta*, *yellow*, and *amber*, all registered 255 (Table 8). Although we were aware of this over-saturation, all color characterization tests of the CI-600 were completed using default settings because the CI-600 encountered systematic communication problems with its software during testing, and we were unable to alter the settings.

**Table 7:** Mean sRGB values (and expanded uncertainties) as measured by the AMR-A.

Color	R	±2SE (R)	G	±2SE (G)	B	±2SE (B)
*Cyan*	0	N/A	107.529	0.059	244.23	0.026
*Magenta*	206.423	0.035	123.219	0.048	200.561	0.041
*Yellow*	232.684	0.022	251.412	0.019	17.079	0.127
*Amber*	231.734	0.024	234.742	0.011	202.499	0.033
*Red*	130.17	0.048	0	N/A	0	N/A
*Green*	0	N/A	89.485	0.061	23.089	0.089
*Blue*	0	N/A	0	N/A	193.347	0.054
*White*	245.703	0.025	255	N/A	255	N/A

Expanded uncertainties represent the repeatability of the camera for the specific color patch. Uncertainties for values at the limits of the sRGB spectrum are not provided. 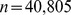 per color patch.

**Table 8:** Mean sRGB values (and expanded uncertainties) as measured by the CI-600.

Color	R	±2SE (R)	G	±2SE (G)	B	±2SE (B)
*Cyan*	90.406	0.026	179.614	0.013	217.403	0.013
*Magenta*	255	N/A	167.971	0.012	200.864	0.011
*Yellow*	255	N/A	249.617	0.013	177.415	0.019
*Amber*	255	N/A	232.644	0.025	214.657	0.033
*Red*	231.103	0.015	95.250	0.027	95.827	0.029
*Green*	87.799	0.024	171.210	0.011	119.531	0.015
*Blue*	78.508	0.047	128.516	0.024	188.291	0.014
*White*	255	N/A	255	N/A	255	N/A

Expanded uncertainties represent the repeatability of the camera for the specific color patch. Uncertainties for values at the limits of the sRGB spectrum are not provided. 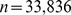 per color patch.

Both minirhizotron cameras had the ability to repeatedly image the same color with a high degree of precision. The largest estimate of AMR-A imprecision was at 0.127, which was at the B vector of the *yellow* color patch. In terms of relative uncertainty, this result equates to ∼0.7%, *i.e*., the signal-to-noise ratio is 99.3∶0.7% (standard error at 95% confidence level). The largest estimate of imprecision in the CI-600, was 0.06% of the signal, which was found at the R vector of the blue color patch. Thus, the signal-to-noise ratio when imaging the eight colors of the specified target was better with the CI-600. But because we did not test over a range of exposures, different lighting etc., we cannot be conclusive if this result is consistent across all colors, or if this is indicative of a population of sensors, but if it is important to the end-user, it should be examined further.

**Figure 4:**
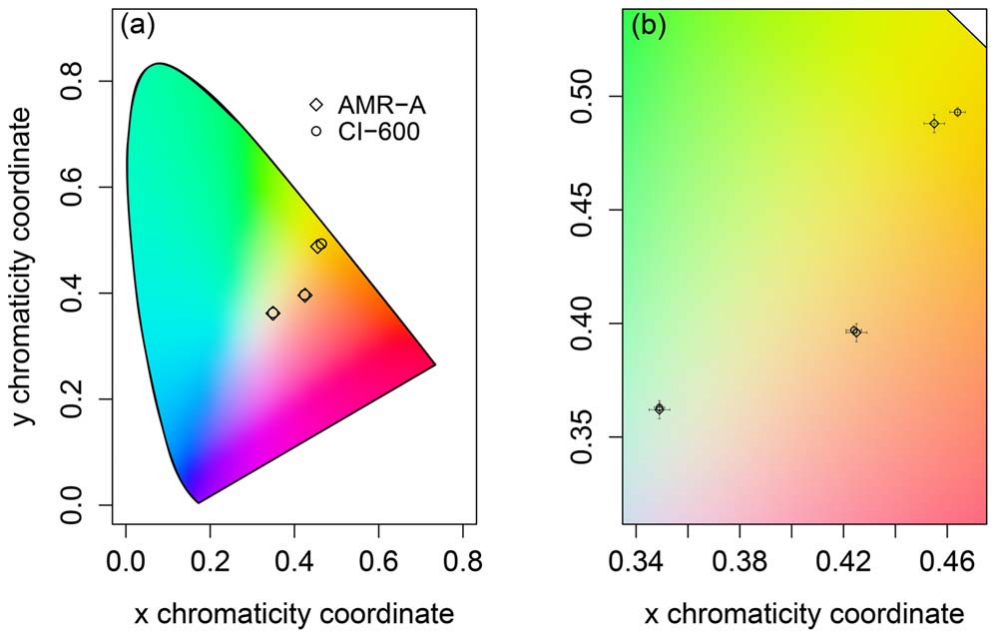
Characterized CIE chromaticity coordinates. CIE chromaticity coordinates of the (a) three characterization points relative to the entire chromaticity coordinate space (as denoted by the horseshoe shaped outline), (b) a zoomed version of the same data in (a) that also depicts the expanded uncertainties of the three characterization points. Error bars are given as ±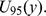 This figure is presented in addition to Table 9 to demonstrate the spatial (color-space) limitations of the linear characterization performed in this analysis. The overlaid color gamut is for illustrative purposes only.

A noted difference among the device-dependent color vectors imaged by the AMR-A and CI-600 was expected. Characterizing the device-dependent sRGB color spaces to the device-independent chromaticity coordinates provided a means by which root colors can be quantified and compared among a group of minirhizotrons with a known accuracy (Table 9; Figure 4). Given the results found here and elsewhere [42], [48]–[50], we recommend that minirhizotron cameras be characterized prior to field deployment with a traceable color test target using the approach presented here and by others [26], [44], [45]. We note that the linear characterization used here may result in different response functions when tested with different cameras (photocells) over a broader color spectrum. The use of colorimetric cameras, and/or a higher order, non-linear characterization, as well as characterizing with a test card comprised of *hundreds* of colors should be explored further [48]–[50]. The resultant coefficients from characterizations should be applied to minirhizotron cameras prior to any type of color analysis. This will allow for traceable quantification of color and will also facilitate estimation of root age and health. We recommend that minirhizotron cameras' are also periodically validated while in the field, as this will aid in quantifying color drift of the camera and photocell degradation over time. Such a task can be completed by installing a color test chart within the minirhizotron tube, preferably at the bottom as to avoid direct interaction with sunlight, and imaging the chart on a periodic basis. Lastly, and possibly most importantly, the natural variation in species-specific relationships between root color and health are likely large, and quite possibly the same magnitude or larger than the variation in color exhibited by these technologies. Only once species and site specific tests are conducted relating root health to chromaticity, do we advise that the classification of root age and or health be defined by chromaticity coordinates. In all cases, however, we highly discourage classifying root age and or health by qualitative, loose nomenclature, such as *white*, *brown*, *cream*, or *black*. This method should also be extended to other camera-based phenological measures and the like.

**Table 9:** Mean, characterized chromatic coordinates (and expanded uncertainties) of each minirhizotron camera.

	AMR-A				CI-600			
Color	x	±U_95_(x)	y	±U_95_(y)	x	±U_95_(x)	y	±U_95_(x)
*Yellow*	0.455	0.004	0.488	0.004	0.464	0.003	0.493	0.002
*Amber*	0.425	0.004	0.396	0.004	0.424	0.003	0.397	0.002
*White*	0.349	0.004	0.362	0.004	0.349	0.002	0.363	0.002

### Heat Dissipation Estimates

**Figure 5:**
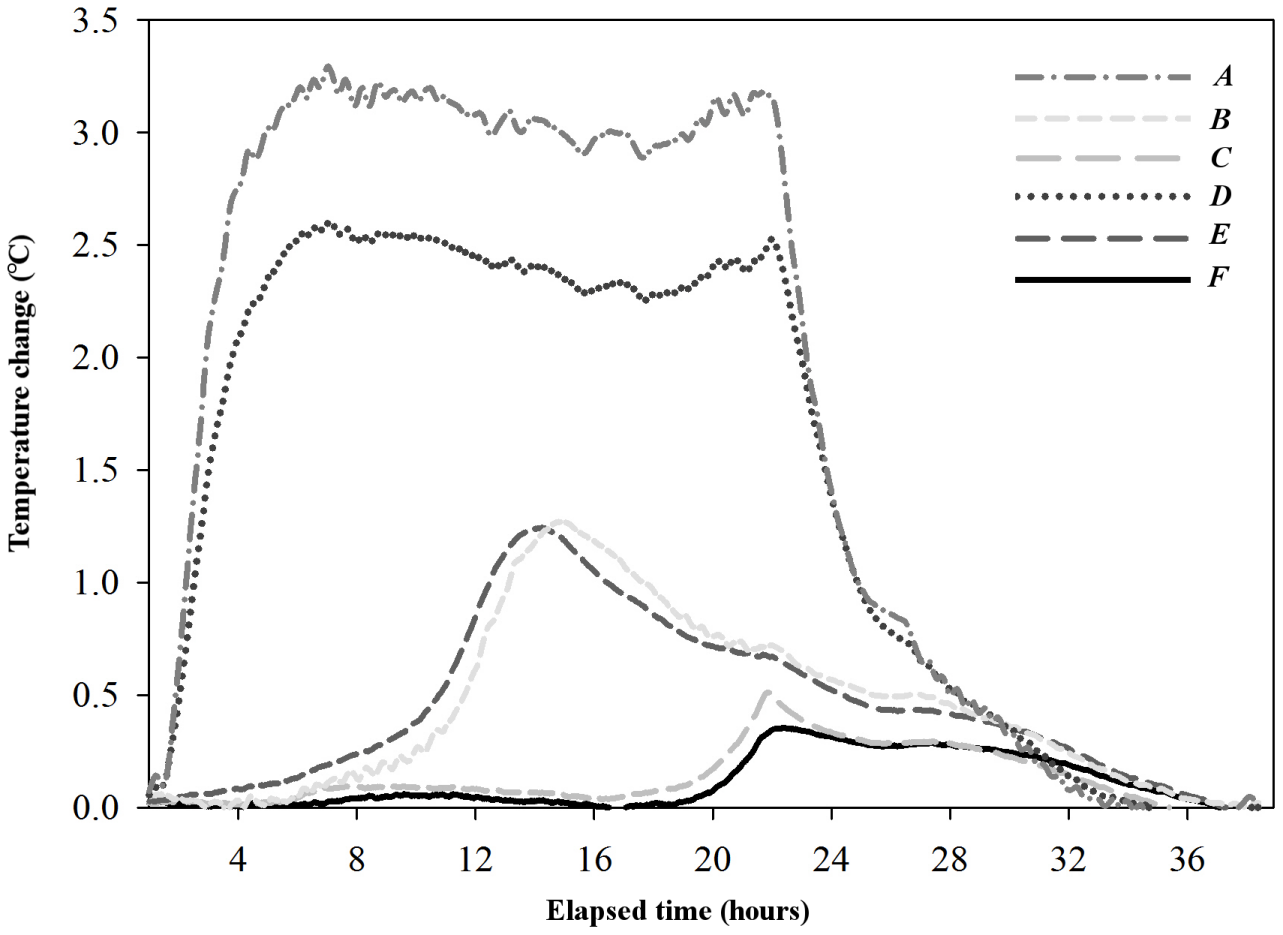
Time series of temperature change at six profile locations. Temperature measurements were made at the three depths (5 [top], 48 [middle], and 86 [bottom] cm) below the surface of the sand and at 0.5 cm away from the AMR-A's tube, the locations of the PRTs *A* through *F* are shown in Figure 2. Temperature change at each location was quantified relative to the temperature of the respective PRT at the start time of each full-tube scan.

When operational, the AMR-A increased temperatures in the environment immediately adjacent to its tube, and away from the tube to a distance of 7.0 cm at all measured depths. These temperature increases, however, were not uniform in time or space. The maximum and mean ambient air temperature increases near the AMR-A's motor (located 10 cm above the sand surface) were 6.74 °C and 3.29±0.05 °C, respectively. Similarly, the maximum and mean ambient air temperature increases near the AMR-A's electrical components (located 30 cm above the sand surface) were 9.36 °C and 7.5±0.05 °C, respectively. The largest temperature increases in the sand were found at shallower depths (*i.e*., closest to the surface; *top* level), *above* (Figure 2), and at a distance of 0.5 cm from the AMR-A's tube (Figure 5). Specifically, the maximum and mean sand temperature increases at this location (point *A*), were 3.25 °C and 1.81±0.02 °C, respectively. The smallest temperature change occurred at the *bottom* level, *below* the AMR-A, and at a distance of 7.0 cm (not shown in Figure 5). The maximum temperature increase at this location was 0.22 °C. However, the mean temperature change at this location was negligible.

The increases in temperatures were not transient, and occurred well before the camera passed a monitored area. Sand temperatures began increasing prior to the camera's passing at PRT locations, and continued to increase for a short period of time after the camera passed. Once reaching their maxima, temperatures persisted above pre-scan values for extended periods of time. For instance, sand temperatures at distances of 0.5 cm away from the AMR-A's tube remained above ambient levels for approximately 28, 23, and 15 hours once maximum temperature change occurred at the *top*, *middle* and *bottom* levels, respectively (Figure 5).

The motor, camera's LED lights, and electrical components of the AMR-A were the major sources of heat. The camera's LED lights, which were attached to the roving camera, were a continuous heat source within the tube. The motor and electronics, which were stationary, were a source of constant heat at the top of the tube. We assumed internal chimney effects within the minirhizotron tube also caused heat to convect to the highest section of tube. Hence, we infer that even if the camera was programmed to image a small and specific region (*i.e.*, not a full tube scan), temperatures generated by the camera's LED lights would rise within the tube, conduct through the AMR-A's tube and warm the surrounding soil.

Clearly, dry sand has a more similar heat capacity to soil than to air, and stores heat similar to that found in soil warming experiments [54], which are designed to alter the soil microclimate in the surrounding rhizosphere. In moist soils, the AMR-A would likely increase the soil vapor pressure and enhance the evaporation of the soil water content around the tube. Given the long operational time it takes the AMR-A to image an entire tube (duration of 25.08±0.43 hours observed during testing; mean ±1 SD), and output of heat from the instrument to the soil, it will likely alter the soil properties, respiration, and likely the measurement of representative root mass (and fine-root turnover) if measurements are conducted frequently, *i.e.*, daily, weekly, monthly or longer pending the ecosystem type.

## Conclusions

Currently, minirhizotrons represent the best non-destructive method for measuring *in-situ* fine-root production, mortality, and turnover. The spatial heterogeneity of fine-root turnover however is typically large [61]–[63], difficult to quantify [64]–[66], and becomes a sample size problem [32], [67]–[69]. Determining seasonal and interannual variability, and long term trends, is a product of matching one's ability to detect a phenomenon with the spatial and temporal signal-to-noise of the phenomenon, in this case fine-root turnover. This is a particularly difficult task because we expect the behavior of fine-roots to change non-linearly in time and space with changes in climate, species composition, nutrient and water availabillty, etc. As we have shown here, if uncalibrated minirhizotrons are used to estimate standing stocks, the risk of improper quantification of fine-root turnover, combined with the amount of undectable roots using scanner-based technologies, have large implications to understand allocation patterns. Knowing the uncertainties of measurements *a priori* is becoming increasingly important as more and more Bayesian and data assimilation approaches are being used in modeling these processes.

Without a traceable and reproducible approach to quantify the measurement uncertainty of minirhizotron camera performance, the signal-to-noise ratio cannot be properly quantified. Once this ratio is quantified, root measurements made by minirhizotrons will be better understood and have increased scientific utilty. Being able to understand and reduce measurement uncertainties in minirhizotrons is key to monitoring root turnover over time and space with minirhizotrons.

Prior to this study no uniform and reproducible method for quantifying and comparing minirhizotron performance existed in the literature. To address this issue, we identified essential, objective, test parameters to assess the minirhizotron cameras' signal-to-noise ratio. We were able to quantify and compare the capabilities of manual and fully automated minirhizotrons to repeatedly image a test target in space, quantify root size and color, and dissipate heat. Because we estimated the signal-to-noise ratio these data can be used as a benchmark to generate sample size requirements given the expected signal-to-noise of fine-root turnover or other ecological processes.

Each minirhizotron camera tested has tradeoffs inherent in their hardware and operating procedures. This included the unavoidable presence of human error in the removal and replacement of the manual minirhizotron camera (CI-600), or the unintended heating of the soil environment (AMR-A). The potential heating effects associated with the AMR-A should, however, be weighed against the much greater sensitivity and repeatability afforded by a fully automated microscopic camera. Additionally, the benefit-to-cost ratio of using a portable camera that can be moved among multiple minirhizotron tubes, such as the CI-600, should be weighed against utilizing a non-portable camera, such as the AMR-A. Regardless, our results suggest that effects of the known, random and systematic uncertainties need to be taken into account when interpreting minirhizotron images and represent an opportunity for new technological advances to increase our knowledge of belowground processes.

In final recommendations; i) one should consider adopting standardized calibration methods (*i.e*., JCGM [26], CIE [45]) of minirhizotron cameras prior to field deployment, and ii) we suggest that known, traceable targets be used in the calibration process and buried with the minirhizotron tubes. Imaging these targets before and after a full-tube scan would help inform the current state of camera health, as the size and color of the targets can be quantified over time and help post correct fine-root data for any optical drift inherent in the camera.

Lastly, the approaches used here are not just germane to minirhizotrons, but to all digital imaging devices used in the scientific process. Approaches and findings presented here have large relevance to other applications, for example, digital microscopy, phenological cameras, digital biodiversity archives, forensics, and others that also intentionally employ device-dependent color imaging.

## Supporting Information

Data S1 **Data and uncertainty analyses for the procedures described in this manuscript.**
(ZIP)
