# Supplementary material for: Traceable Calibration, Performance Metrics, and Uncertainty Estimates of Minirhizotron Digital Imagery for Fine-Root Measurements
Source: PLoS One. 2014 Nov 12;9(11):e112362. doi: 10.1371/journal.pone.0112362 (PMC4229195; doi:10.1371/journal.pone.0112362)
Supplement: Data S1 — Data and uncertainty analyses for the procedures described in this manuscript. (ZIP) [file pone.0112362.s001.zip › Data for submission/Sensitivity and Root Diameter/Micrometer line widths.pdf]

1653 East Main Street
Rochester, NY 14609 USA
Voice: 585.482.0300
FAX: 585.288.5989
imaging@appliedimage.com

Calibration Data
PN QA-60-TL5003(53716)

APPLIED® IMAGE Inc

**Part Number:**	QA-60-TL5003 (53716)	**Job Number:**	7341-2
**Serial Number:**	7341-2	**Job Name:**	National Ecological Observatory Network
**Calibration Date:**	3/7/2014	**Temperature:**	66.0°F
**Operator:**	BJM	**Relative Humidity:**	33%

Instructions: On the vertical line wedge pattern, measure each line width of the left bar (first line) PERPENDICULAR to left-side of left-most line and in line with label indicator line – see image example. Add serial number on backside lower-right corner.

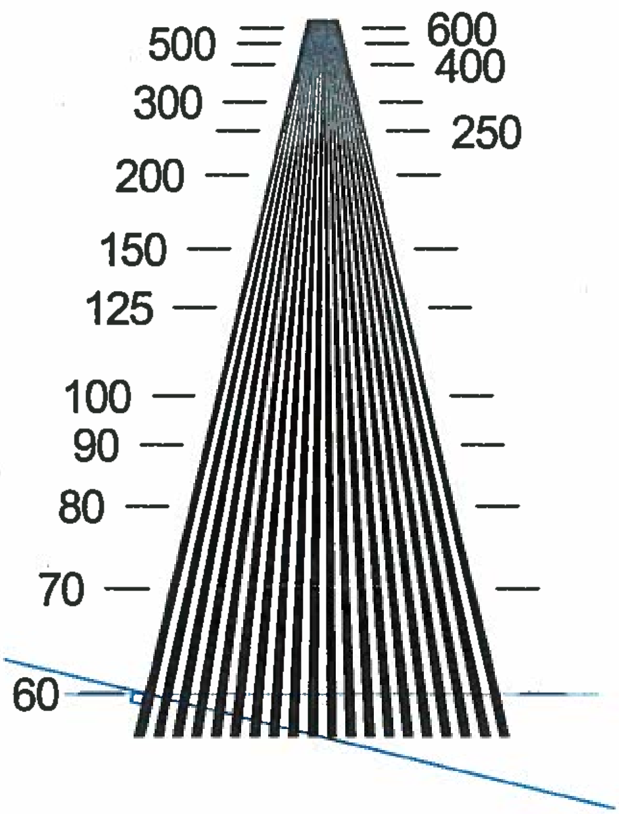

LPI		Nominal line widths (inch)		As measured (inch)
60		0.016667		0.016127
70		0.014285		0.013709
80		0.012500		0.011950
90		0.011111		0.010570
100		0.010000		0.009552
125		0.008000		0.007640
150		0.006667		0.006376
200		0.005000		0.004735
250		0.004000		0.003943
300		0.003333		0.002906
400		0.002500		0.002283
500		0.002000		0.001719
600		0.001667		0.001547

Precision IMAGE Products & Services

1653 East Main Street
Rochester, NY 14609 USA
Voice: 585.482.0300
FAX: 585.288.5989
imaging@appliedimage.com

Calibration Certificate
Linear Dimensions

APPLIED ® IMAGE Inc

**Part Number:**	QA-60-TL5003 (53716)	**Test Report/ Job #:**	7341-2
**Serial/ ID/ Calib #:**	7241-2	**Client Name:**	National Ecological Observatory Network
**Certification Date:**	3/7/2014	**Client Address:**	1685 38th Street suite 100 Boulder ,CO 80301 USA
**Part Description:**	Kodak Digital Science Imaging Target	**Purchase Order #:**	CC 2/27/14
**Method of Measurement:**	Filar (AI206)		

These parts are inspected on equipment calibrated with standards traceable to the National Institute of Standards and Technology, USA.

The results of this calibration apply only to the serial number of the part stated on this document. This calibration certificate shall not be reproduced, except in full, without written approval of Applied Image, Inc.

Equipment Used In Calibration	Estimated Uncertainty	Traceable to NIST via NIST Test #:
AI-206 400x Filar 0 - 200 µ	0.69µ	821/253660-94
AI-206 200x Filar 201– 400µ	0.82µ	821/253660-94
AI-206 100x Filar 401– 800µ	1.11µ	821/253660-94

The above estimated uncertainties are based on the measurement of stable base items with sharp image edges.

Name: BJ McCutchan

Function: Quality Assurance

Signature: BJ McCutchan

Notes:
