# Supplementary material for: Traceable Calibration, Performance Metrics, and Uncertainty Estimates of Minirhizotron Digital Imagery for Fine-Root Measurements
Source: PLoS One. 2014 Nov 12;9(11):e112362. doi: 10.1371/journal.pone.0112362 (PMC4229195; doi:10.1371/journal.pone.0112362)
Supplement: Data S1 — Data and uncertainty analyses for the procedures described in this manuscript. (ZIP) [file pone.0112362.s001.zip › Data for submission/Root Color/SO 7341-1 CALIBRATION CERTIFICATE AI-314 0-45 SPECTRAL REFLECTION rev1-04.doc]

These parts are inspected on equipment calibrated with standards traceable

to the National Institute of Standards and Technology, USA.

Results of this calibration apply only to the serial number of the part stated on this document.

This calibration certificate shall not be reproduced, except in full, without written approval of Applied Image, Inc.

| Equipment Used  In Calibration: | AI-314 | Traceable to  NIST via: | Certificate #MCSL-20089022-1  NRC Test #PAR-2007-2528 |
| --- | --- | --- | --- |

| **Spectral Reflectance** | | | | | |
| --- | --- | --- | --- | --- | --- |
| The following uncertainties for Spectral Reflectance are calculated for near neutral reflectances  (ie: whites, greys and blacks) | | | | | |
| % Reflectance Range | 100% to 45%R | 44.9% to 15%R | 14.9% to 4%R | 3.9% to 1.0%R | 0.99% to 0%R |
| Spectral Range | Expanded Uncertainty (~95%, k=2) plus max potential range error | Expanded Uncertainty (~95%, k=2) plus max potential range error | Expanded Uncertainty (~95%, k=2) plus max potential range error | Expanded Uncertainty (~95%, k=2) plus max potential range error | Expanded Uncertainty (~95%, k=2) plus max potential range error |
| 400-419nm | 0.526 + 2.2% R | 0.223 + 1.32% R | 0.222 + 0.44% R | 0.030 + 0.21% R | 0.042 + 0.17% R |
| 420-450nm | 0.525 + 0.78% R | 0.223 + 0.45% R | 0.221 + 0.14% R | 0.026 + 0.08% R | 0.037 + 0.09% R |
| 451-700nm | 0.511 + 0.92% R | 0.222 + 0.56% R | 0.221 + 0.15% R | 0.024 + 0.13% R | 0.037 + 0.11% R |

| **CIE L*a*b*** | | | | | | | | | |
| --- | --- | --- | --- | --- | --- | --- | --- | --- | --- |
|  | CIE L*a*b* measurements: Instrument short term typical repeatability (n=30)  D50/ 2 degree Illumination conditions: M0 | | | | | | | | |
|  | White | | | Blue | | | Brown | | |
|  | L* | a* | b* | L* | a* | b* | L* | a* | b* |
| CIE L*a*b* value | 94.515 | -1.139 | 0.383 | 28.903 | 8.389 | 3.881 | 32.148 | 36.702 | 8.714 |
| 2 sigma | 0.025 | 0.022 | 0.011 | 0.016 | 0.028 | 0.037 | 0.015 | 0.024 | 0.092 |
| Range | 0.050 | 0.040 | 0.020 | 0.030 | 0.060 | 0.080 | 0.030 | 0.050 | 0.190 |
| delta E (1976) | 0.067 | | | 0.104 | | | 0.199 | | |

Reported CIE L*a*b* values are calculated directly from spectral data (see spectral reflectance table for uncertainties). The above values are based on measurements from white, blue, and brown test patches of a Xrite P/N968-62 porcelain on steel test plaque.

Note that it is very common for color surfaces to exhibit L*, a*, and/or b* variations within test areas of 1.0 or higher (10 to 50 times higher than the typical instrument repeatability), so this will likely be the main cause of variation for most users.

(2 sigma is 2 standard deviations, Range is the difference between the highest and lowest measured value, Delta E (1976) is calculated from the differences in worst case repeat measurements (i.e.; Range))

Page 1 of 2

| Equipment Used  In Calibration: | AI-314 | Traceable to  NIST via: | Certificate #MCSL-20089022-1  NRC Test #PAR-2007-2528  (secondary Status A density check performed using artifact traceable to :NIST SRM #1930-90 |
| --- | --- | --- | --- |

| Status A Reflection Density (ANSI PH 2.18, ISO 5/3) | | | | |
| --- | --- | --- | --- | --- |
|  | Filter:Visual (V) | Filter: Red (C) | Filter: Green (M) | Filter: Blue (Y) |
| Reflection Density Range | Expanded Uncertainty (~95%, k=2) plus max potential range error | Expanded Uncertainty (~95%, k=2) plus max potential range error | Expanded Uncertainty (~95%, k=2) plus max potential range error | Expanded Uncertainty (~95%, k=2) plus max potential range error |
| 0 - 0.84D | 0.0061 +0.003D | 0.0061 + - 0.003D | 0.0061 + 0.001D | 0.0061 + -0.008D |
| 0.841 - 1.5D | 0.0061 + 0.004D | 0.0061 + 0.003D | 0.0061 + 0.003D | 0.0061 + -0.004D |
| 1.51 - 1.95D | 0.0062 + 0.022D | 0.0064 + 0.020D | 0.0064 + 0.018D | 0.0071 + 0.018D |
| 1.951 - 2.3D | 0.0070 + 0.037D | 0.0076 + 0.037D | 0.0077 + 0.040D | 0.0105 + -0.131D |

| Name: | David Curtis |  |
| --- | --- | --- |
| Function: | Quality Assurance |  |
| Signature: |  |  |

input.doc	Page 2 of 2

Notes:
