# Supplementary material for: Traceable Calibration, Performance Metrics, and Uncertainty Estimates of Minirhizotron Digital Imagery for Fine-Root Measurements
Source: PLoS One. 2014 Nov 12;9(11):e112362. doi: 10.1371/journal.pone.0112362 (PMC4229195; doi:10.1371/journal.pone.0112362)
Supplement: Data S1 — Data and uncertainty analyses for the procedures described in this manuscript. (ZIP) [file pone.0112362.s001.zip › Data for submission/Root Color/SO7341-1_QA-69 Cal Form.doc]

**Instructions:** Measure center and 4 corners of each color patch in CIE L*a*b* D50/2 degrees conditions. Report 5 measurements per color, plus Average CIE L*a*b* values. Record and save Spectral Reflectance data for each of the 5 measurements per patch, save to CD to send to customer. Add serial number to back side, lower-right corner.

| **#** | **Patch** | **Nominal CIE L*a*b*** | |  | **Read 1** | **Read 2** | **Read 3** | **Read 4** | **Read 5** | **AVG. read** |
| --- | --- | --- | --- | --- | --- | --- | --- | --- | --- | --- |
| **1** | **Cyan** | **L*** | **50.61** |  | 49.08 | 49.05 | 48.86 | 48.93 | 48.86 | 48.96 |
|  |  | **a*** | **-9.92** |  | -22.60 | -22.53 | -22.40 | -22.42 | -22.49 | -22.49 |
|  |  | **b*** | **-47.89** |  | -50.82 | -50.74 | -50.66 | -50.57 | -50.69 | -50.69 |
|  |  |  |  |  |  |  |  |  |  |  |
| **2** | **Magenta** | **L*** | **64.56** |  | 63.99 | 64.11 | 64.06 | 63.91 | 64.06 | 64.03 |
|  |  | **a*** | **50.02** |  | 56.76 | 56.57 | 56.51 | 56.34 | 56.33 | 56.60 |
|  |  | **b*** | **-17.81** |  | -6.05 | -6.09 | -5.89 | -6.00 | -5.93 | -5.99 |
|  |  |  |  |  |  |  |  |  |  |  |
| **3** | **Yellow** | **L*** | **86.93** |  | 89.05 | 89.12 | 89.17 | 89.09 | 89.04 | 89.09 |
|  |  | **a*** | **-8.97** |  | -3.94 | -3.89 | -3.84 | -3.82 | -3.79 | -3.86 |
|  |  | **b*** | **95.77** |  | 95.85 | 95.25 | 95.71 | 95.36 | 95.24 | 95.48 |
|  |  |  |  |  |  |  |  |  |  |  |
| **4** | **Amber** | **L*** | **84.68** |  | 83.72 | 84.35 | 84.40 | 84.25 | 84.36 | 84.22 |
|  |  | **a*** | **17.47** |  | 15.03 | 15.22 | 15.18 | 15.37 | 15.40 | 15.24 |
|  |  | **b*** | **28.55** |  | 31.01 | 31.42 | 31.50 | 31.31 | 31.46 | 31.34 |

| **#** | **Patch** | **Nominal CIE L*a*b*** | |  | **Read 1** | **Read 2** | **Read 3** | **Read 4** | **Read 5** | **AVG. read** |
| --- | --- | --- | --- | --- | --- | --- | --- | --- | --- | --- |
|  |  |  |  |  |  |  |  |  |  |  |
| **5** | **Red** | **L*** | **39.97** |  | 36.37 | 36.22 | 36.25 | 36.30 | 36.18 | 36.26 |
|  |  | **a*** | **66.95** |  | 67.84 | 67.93 | 68.06 | 67.62 | 68.00 | 67.89 |
|  |  | **b*** | **49.36** |  | 47.69 | 48.32 | 48.26 | 47.02 | 48.44 | 47.95 |
|  |  |  |  |  |  |  |  |  |  |  |
| **6** | **Green** | **L*** | **47.42** |  | 46.36 | 46.45 | 46.57 | 46.40 | 46.35 | 46.43 |
|  |  | **a*** | **-52.54** |  | -59.14 | -59.68 | -59.40 | -58.99 | -59.07 | -59.26 |
|  |  | **b*** | **24.65** |  | 22.90 | 23.19 | 23.05 | 22.82 | 22.92 | 22.98 |
|  |  |  |  |  |  |  |  |  |  |  |
| **7** | **Blue** | **L*** | **31.04** |  | 25.46 | 25.37 | 25.53 | 25.42 | 25.70 | 25.49 |
|  |  | **a*** | **14.19** |  | 2.12 | 2.14 | 1.93 | 2.05 | 2.13 | 2.07 |
|  |  | **b*** | **-53.96** |  | -59.73 | -59.86 | -59.87 | -59.90 | -59.61 | -59.80 |
|  |  |  |  |  |  |  |  |  |  |  |
| **8** | **White** | **L*** | **96.65** |  | 94.67 | 94.80 | 94.84 | 94.56 | 94.97 | 94.77 |
|  |  | **a*** | **-1.41** |  | -0.36 | -0.31 | -0.30 | -0.34 | -0.29 | -0.32 |
|  |  | **b*** | **4.10** |  | 2.15 | 2.30 | 2.23 | 2.18 | 2.35 | 2.24 |
